# SARS-CoV-2 infection: a global outbreak and its implication on public health

**DOI:** 10.1186/s42269-021-00599-7

**Published:** 2021-08-03

**Authors:** Sankari Mohan, M. Reshma Anjum, Anusha Kodidasu, T. V. N. Sai Prathyusha, Nunna Venkata Mrunalini, B. Kishori

**Affiliations:** Sri Padmavathi Mahila Visvavidyalayam (Women’s University), Tirupati, Andhra Pradesh India

**Keywords:** COVID-19, Pandemic, SARS-CoV-2019, ACE2 receptors, Structural proteins

## Abstract

**Background:**

A novel corona virus is formally named as severe acute respiratory syndrome coronavirus 2 (SARS-CoV-2), which results in causing coronavirus disease 2019 (COVID-19). It is the latest prevalent pandemic worldwide when compared to other infectious diseases like Avian flu, Middle East respiratory syndrome and severe acute respiratory syndrome (SARS).

**Main body:**

Coronavirus disease 2019 (COVID-19) is currently occurring pandemic over world. It was emerged in Wuhan, China, in the end of December 2019 and spreading across worldwide. As the coronavirus is spreading easily through direct contact with infected people droplets, inhalation, and also air droplets, it hit up a huge amount of population even reported with death. Still, with small amounts of asymptomatic transmission between people it spreads throughout the globe. People need special care to protect from the transmission of disease. However, there are no drugs so far that shows efficacy; there is an immediate need for the development of vaccines. In order to decrease the COVID-19 cases, organizations rapidly involve in the preparation of vaccine and many vaccines have been developed by various countries. The governments took safety measures to control the spread of virus and also to minimize morbidity and mortality rate to least possible.

**Conclusion:**

The purpose of this review article is to increase our understanding of COVID-19 and facilitate the people to take a move in facing challenges of the world.

## Background

A novel corona virus is formally named as severe acute respiratory syndrome coronavirus 2 (SARS-CoV-2), which results in causing coronavirus disease 2019 (COVID-19). According to WHO (World Health Organisation), it is the latest prevalent pandemic worldwide when compared to other infectious diseases like Avian flu, Middle East respiratory syndrome (MERS) and severe acute respiratory syndrome (SARS). It was initially recognized in December 2019 in Wuhan, Hubei, China (Li 2004; Hui et al. 2020). This new disease is caused by a new strain in the corona virus family that even infects birds and mammalians. Before this virus, six different corona viruses which infect humans and mainly cause respiratory tract infections were identified. Among them, two cause MERS and SARS, respectively (Raoult et al. 2020). WHO declared this COVID-19 a pandemic! which means the disease happening in wide range of geographical area and affecting large number of populations. WHO declared the coronavirus disease named as COVID-19 based on CO referring to corona, VI to virus, and D to disease and 19 to 2019 on February 11; then the first case was known at the end of 2019. International Committee on Taxonomy of Viruses (ICTV) named the virus as “severe acute respiratory syndrome coronavirus 2 (SARS-CoV-2)” because the viruses have some genetic relation to SARS outbreak in 2003.

SARS-CoV-2 passed over to humans at one of Wuhan’s open-air “wet markets”, and it is the place where customers buy fresh meat and fish, including animals that were killed on the spot. These crowded conditions let viruses swap genes from different animals. Corona viruses that are similar to SARS-CoV-2 infect the pangolins (Liu et al. 2020). The researches concluded that SARS-CoV-2 made a possible jump by exchanging an essential fragment of a gene in the virus in pangolins, and it spreads to the people who had consume it. As of 17 June 2021, there have been over 3,851,704 deaths due to 2019-nCoV/SARS-CoV-2 outbreak worldwide (WHO), which concludes that human-to-human transmission is possible among the close contacts (Li et al. 2020a, b). Scientists discovered first human coronavirus in 1965 and that caused a common cold. In China, they found two different strains in a study of 103 COVID-19 cases, where they named it as L type which was most common in early stage of outbreak and S type is older (Tang et al. 2020).

Currently, most of the national and international researchers are working on the progress of vaccines to stop and treat the 2019-nCoV/SARS-CoV-2, but there are no effective medicines against 2019-nCoV/SARS-CoV-2. There is an immediate need for the development of effectual prevention and powerful treatment strategies for 2019-nCoV/SARS-CoV-2 outbreak (Zhou et al. 2020).

## Main text

### Structural organization of corona virus

Corona viruses (CoVs) are enveloped, +ssRNA viruses and hold an unusual large RNA genome and have unique replication strategy. Corona viruses cause a variety of diseases in animals ranging from cows, pigs and birds and also use several intermediate hosts such as ranging from snakes, pangolins, turtles and other wild animals to humans which causes potentially lethal respiratory infections in humans. Hence, these viruses can efficiently use both animal and human hosts (Tiwari et al. 2020). Corona virus is one of the largest RNA virus with 80–160 nm in diameter and the genome ranges from 26 -34 kilo bases (Bar-on et al. 2020) and also the virus particles are typically ~ 20 nm and have club- or petal-shaped surface projections called "peplomers" or "spikes" (Masters 2006). Common features of corona viruses contain conserved genomic organization with a large replicase gene which contains structural and accessory genes. Moreover, non-structural genes can be expressed by ribosomal frame shifting along with numerous unique unusual enzymatic activities encoded with the aid of large replicase-transcriptase polyprotein and also expression of downstream genes by synthesis of 3’-nested sub–genomic mRNAs (Gaurav and Al-Nema 2019).

SARS-CoV-2 is a single-stranded positive-sense RNA genome encodes 10 genes which produces 26 proteins based on NCBI database. It contains one long gene, orf1ab, codes a polyprotein which cleaved into 16 proteins by proteases enzyme and act as a part of a polyprotein which encodes an RNA polymerase and associated factors, a proof-reading exonuclease and some non-structural proteins (Bar-on et al. 2020). The structural components of the corona virus are the spike (S) protein that binds to the specific receptor on an animal or human host cell, nucleoprotein (N) that packages the genome, envelope (E) and membrane (M) proteins are two membrane-bound proteins (De Haan et al. 2000; de Haan and Rottier 2005; Perlman and Netland 2009). Various current works are going on to understand the role of 'Accessory' proteins in the viral life cycle (Bar-on et al. 2020; Artika et al. 2020). With these four structural proteins, some corona viruses have the fifth structural protein called as hemagglutinin esterase (HE) protein. All these proteins of SARS-CoV-2 are vital for efficient assembly, trafficking, and release of virus-like particles throughout viral life cycle (Siu et al. 2008).

Spike (S) proteins are trimeric, glycoproteinaceous in nature with high molecular weight (Bosch et al. 2005). These spike proteins along with HE protein assist in viral entry into the human cell. It comprises a “receptor-binding domain”, which recognizes a specific receptor named as “angiotensin-converting enzyme receptor 2 (ACE2)”. These receptors were expressed in host lungs, heart, kidneys and intestine cells. It was detected that the protein quandaries to ACE2 receptor with great potentiality than the SARS virus, which shows the reason behind fast and efficient spreading of virus. Spike proteins have two functional subunits: S1 which binds to the host cell receptor and S2 which mediates the internal fusion of the virus with the host cellular membranes (Belouzard et al. 2012). These two sub-proteins play a major role in binding and cellular entry into the target cells of the host (Ortega et al. 2020).

Nucleocapsid (N) is multifunctional proteins and contains 46 kDa protein composed of 422 amino acids. It consists of N-terminal region which is responsible for RNA binding and C-terminal region responsible for nuclear localization signal (Rota et al. 2003; Surjit and Lal 2008). The main function of N-protein is to bind the CoV-RNA genome, which makes up the nucleocapsid. Even though N protein is largely complicated in functional process, it is associted with viral genome, CoV replication cycle and also involved in host cellular response to viral infection. Stimulatingly, N proteins localization from the endoplasmic reticulum (ER) to Golgi region (GC) has projected a role for its assembly and budding. However, transient expression of N protein showed a substantial increase in the production of virus-like particles (VLPs) in some CoVs, suggesting it might not be required for envelope formation, but necessary for complete virion formation (De Haan et al. 2020; McBride et al. 2014).

Envelope (E) proteins help in the formation of viral envelope. These proteins play numerous roles in virus infection and pathogenesis. An E protein majorly helps in viral morphology because these are the front-line components in viral envelope formation. E proteins contains 74–109 amino acids with hydrophobic viroporins (Hogue and Machamer 2007; Wilson et al. 2004). It contains two distinct structural domains such as a longer typical hydrophobic domain and a charged cytoplasmic tail. These envelope proteins are two different types like E1 and E2 proteins. E1 proteins form the trans-membrane glycoprotein matrix, whereas the E2 proteins form the peplogenic glycoproteins which are important in fusion of viral particles after replication. These proteins act as a site of intercellular trafficking during viral replication more specifically at Golgi apparatus and endoplasmic reticulum. The requirement for E proteins during virus morphogenesis diverges depending on the virus genus (Venkatagopalan et al. 2015).

Membrane (M) proteins are the most abundant proteins on the viral surface, and this expresses the shape of the viral envelope (De Haan et al. 2020). The M protein of corona virus plays a key role in assembly of viral particles and turning cellular membranes into workshop, where the virus and host factors originate together to make novel virus particles. M-protein helps assembly of viral ribonucleoprotein and S glycoprotein at budding site, and it forms an M–M interaction network having the capable of excluding the host membrane proteins from the viral envelope (De Haan et al. 2020; Neuman et al. 2008). M protein also cooperates with RNA that conveys the genomic packaging signal (Narayanan et al. 2003). M protein adopts two conformations in which the membrane curvature is controlled by one M conformer, and compact M protein is connected with different conformation such as flexibility and low spike density. The elongated M protein is connected with rigidity, relatively narrow range of membrane curvature and cluster of spikes (Neuman et al. 2011). Investigation of some types of virus-like particles and virions exposed that S protein, N protein and genomic RNA each aid to regulate virion size and variation, presumably through interactions with M. When compared to all structural proteins, M and E proteins are very essential for viral replication and pathogenesis. These findings provide awareness into how M protein functions to promote virus assembly.

Recently, the whole world is getting alert towards the new strain of SARS-CoV-2 which is responsible for COVID-19 disease. Over 10,000 COVID cases in India were sequenced and discovered a new strain. This is because of mutations because the viruses are prone to it. Viruses undergo mutations to make them the survival of fittest. 15–20% of samples from Maharashtra in India show two new mutations in key areas of viruses. These mutations are called as E484Q and L452R. These mutations along with P681R were concerned because the mutations took place at the site of spike protein which may possibly increase ACE2 receptor binding. The mutations in the spike protein made them to have easy entry and faster multiplication (Cherian et al. 2021).

Most widely talked mutation is D614G variant. This mutation causes change in spike protein. An amino acid at position 614 in spike protein changed from aspartic acid to glycine. This mutant form was observed in China and then in Europe. Some researchers said that these changes make the variant to infect people more easily because this mutation is located in the subunit S1 of spike protein. This D614G variant becomes the most predominant one of SARS-CoV-2 worldwide. B.1.1.7 variant undergoes 23 mutations, where 6 of them have no amino acid change and in other 17 mutations, 8 having effect on spike protein. N501Y variant is the one in which the amino acid asparagine is changed to tyrosine at position 501 located on spike protein. This variant was observed in UK which is believed to be the most transmissible than the wild type (Elbe and Buckland‐Merrett 2017; Shang et al. 2020). A list of different SARS-CoV variants occurring globally is given in Table 1. However, every country saw a decrease in the cases in the mid-October, but there is a sudden increase in cases. This is because the new variants have capability of spreading fast and also have affective towards the humans.

**Table 1:** A list of different SARS-CoV-2 variants that occurred globally

COVID variant	Substitutions in spike protein	Origin	Characteristics	References
B.1.1.7	Δ69/70, Δ144, N501Y, A570D, D614G, P681H, T716I, S982A, D1118H	UK	Approx. 50% transmission No impact with monoclonal antibody treatment	Wang et al. (2021)
B.1.351	D80A, D215G, Δ241/242/243, K417N, E484K, N501Y, D614G, A701V	South Africa	Approx. 50% transmission Decrease with combination of bamlanivimab and etesevimab and monoclonal antibody treatment	Wu et al. (2021), Wang et al. (2021)
B.1.427	L452R, D614G	USA	Approx. 20% transmission Less chance of decrease with combination of bamlanivimab and etesevimab and monoclonal antibody treatment	Zhou et al. (2021)
B.1.429	S13I, W152C, L452R, D614G	USA	Approx. 20% transmission Less chance of decrease with combination of bamlanivimab and etesevimab and monoclonal antibody treatment	Zhou et al. (2021)
P.1	L18F, T20N, P26S, D138Y, R190S, K417T, E484K, N501Y, D614G, H655Y, T1027I	Brazil and Japan	Decrease with combination of bamlanivimab and etesevimab and monoclonal antibody treatment	Zhou et al. (2021)
B.1.526	T95I, D253G, D614G	USA	Decrease with combination of bamlanivimab and etesevimab and monoclonal antibody treatment	Annavajhala et al. (2021)
B.1.526.1	Spike: D80G, Δ144, F157S, L452R, D614G, (T791I*), (T859N*), D950H	USA	Reduction in neutralization by some EUA monoclonal antibody treatments	Annavajhala et al. (2021)
B.1.525	Spike: A67V, Δ69/70, Δ144, E484K, D614G, Q677H, F888L	UK/Nigeria	EUA monoclonal antibody treatments	Zhou et al. (2021)
P.2	Spike: E484K, (F565L*), D614G, V1176F	Brazil	EUA monoclonal antibody treatments	Zhou et al. (2021)
B.1.617	Spike: L452R, E484Q, D614G	India	Potential reduction in neutralization by some EUA monoclonal antibody treatments	Yadav et al. (2021)
B.1.617.1	Spike: (T95I), G142D, E154K, L452R, E484Q, D614G, P681R, Q1071H	India	EUA monoclonal antibody treatments	Yadav et al. (2021)
B.1.617.2	Spike: T19R, (G142D), Δ156, Δ157, R158G, L452R, T478K, D614G, P681R, D950N	India	EUA monoclonal antibody treatments	Yadav et al. (2021)
B.1.617.3	Spike: T19R, G142D, L452R, E484Q, D614G, P681R, D950N	India	EUA monoclonal antibody treatments	Yadav et al. (2021)

### Life process

The corona virus life cycle within the host consists of 5 steps; they are attachment, penetration, biosynthesis, maturation and release. If the viruses are attached to host receptors, they penetrate into the host cell via endocytosis or membrane fusion. After the viral genome is delivered inside the host cell it  passes through the nucleus of host cell for replication and  initiates viral biosynthesis. After protein biosynthesis, new viral particles are matured and come through out of the cell via cell lysis (Yuki et al. 2000; Bosch et al. 2003).

Corona virus spike protein helps in adhering and entry into target host cells. Angiotensin-converting enzyme 2 (ACE2) acts as receptor for SARS-CoV and SARS-CoV-2 (Hoffmann et al. 2020). The entry of the SARS-CoV-2 to the host cell is self-regulating of ACE2 catalytic activity, and entry is mediated through 2 spike protein subunits; for example, S1 facilitates through ACE2 attachment via the receptor-binding domain and S2 subunit contains fusion peptide and trans-membrane domain. These subunits involve in the fusion of host and viral particles (Lan et al. 2020). For activation of fusion, the spike protein should be cleaved at 2 sites directly through endosomes at the cell membrane. The cleavage site sequences were located at the border of S1, S2 subunits, and the other was located within the upstream of the fusion peptide which give substrate for cellular proteases and cleavage efficiency determination (Silverman 2020).

The direction of the infection is depending on the presence of protease enzyme in different cell types and the protease cleavage sites. This was confirmed by activity of cellular serine protease TMPRSS2 (Transmembrane serine protease 2), furin and endosomal cathepsins B and L in SARS-CoV-2 entry (Millet and Whittaker 2015). The host protease enzymes which cleave the S protein play a major role in antiviral drugs (Lan et al. 2020). For replication of viral genome, first viral RNA genome has passed through the cytoplasm and is translated into two polyproteins and structural proteins. The newly formed envelope glycoproteins are inserted into the membrane of the endoplasmic reticulum or Golgi, and the nucleocapsid is formed by the combination of genomic RNA and nucleocapsid protein. Then, viral particles germinate into the endoplasmic reticulum-Golgi intermediate compartment (ERGIC). At last, the vesicles containing the virus particles then fuse with the plasma membrane to release (Li et al. 2020a, b). After producing the sufficient amounts of new genomic RNA and structural proteins, assembly of particles occurs. Assembly and release of virions are the last stages of the virus life cycle (Siu et al. 2008).

### Few pandemics with their epidemiology

COVID-19 came into light in the year 2019 caused by a novel virus SARS-CoV-2. The first case was observed in Wuhan city, China, in the month of December. Transmission is mainly through respiratory droplets, human-to-human and also through touching the contaminated surfaces. Symptoms include common cold, chills, cough, improper breathing, kidney failure finally leads to death (Harapan et al. 2020; Lotfi et al. 2020), whereas Middle East respiratory syndrome (MERS) that belongs to the same family of Coronaviridae was first observed in the year 2012 in Saudi Arabia. It transmits mainly through person-to-person. However, the symptoms are same as the novel SARS-CoV-2 as it belongs to the same family (Ramadan and Shaib 2019). In mid-April 2009, a virus named “H1N1 influenza virus (nH1N1) or Swine-Origin Influenza Virus” (S-OIV) was identified in Mexico City; later, it was named as ‘swine–flu” and is very contagious (Gibbs et al. 2009). As this swine-flu is a subtype of Influenza A, the symptoms are also similar to those of influenza like fever, body pains, raw throat, extreme fatigue (Dandagi and Byahatti 2011). SARS (severe acute respiratory syndrome) outbreak was first observed in Foshan city, Guangdong province, China, 16 November 2002 caused by the virus SARS-CoV (Xu et al. 2004). As it belongs to the family Coronaviridae, the transmission modes and symptoms are similar to MERS and SARS-CoV-2. Ebola viral disease (EVD) was first identified in 1976 near the Ebola River and called Democratic Republic of Congo. It belongs to the family Filoviridae. This virus infects people who had contacted with infected body fluids leading to outbreaks in several African countries (Kourtis et al. 2015). In 1957–1958, influenza pandemic occurred globally which was also known as the Asian flu pandemic that was caused by “Influenza A virus subtype H2N2″ which was identified in East Asia. This spreads rapidly, and there is no maximal incidence to provide good evidences for the routes of spread (Jordan Jr 1961). Lesser symptoms are like chills and cough, whereas extreme symptom is like pneumonia. Another flu called Spanish flu (1918 influenza pandemic) spreads throughout the world during First World War. This pandemic was caused by the virus H1N1 Influenza A Virus. This disease shows flu like symptoms and spreads through respiratory droplets in first wave, whereas the second wave shows severe symptoms which are highly communicable and the high death rate was at peak. The third wave comes and also exists but with fewer deaths (Table 1) (Martini et al. 2019).

### Viruses causing major outbreaks

Humans had been defending with the viruses from past many decades. Several years ago, vaccines and antiviral drugs were used to keep us safe from infections that were spreading widely and had helped several people getting sick from some viral diseases. And we lost in defeating against viruses from winning. This is because the viruses underwent several genetical changes and move from animals to mankind resulting in killing many of the lives that leads to outbreaks. These outbreaks further lead to pandemics. Pandemics are said to be large size eruptions which usually caused by infective agents leaving in huge death rate that completely affect the socio-economic ratio. However, pandemics have become the most common because of increasing social process whereby cities grow and societies become more urban, altering in the layers of the surface of the earth and also exploiting the surroundings (Jones et al. 2008; Morse 2001). A pandemic was determined as “an outbreak that spreads to vast geographical region of indefinite boundary leading to high death ratio” (Porta 2014). Hence, pandemics were identified based on the spreading but not on extreme unwellness. Almost all pandemics are of zoonotic origin (Murphy 1998; Woolhouse and Gowtage-Sequeria 2005). Here, Table 1 lists out some biggest experienced outbreaks caused by many infective agents.

### Vaccines for prevention—past, present and future

Edward Jenner, Louis Pasteur and other pioneers discovered “vaccination” several hundred years ago and determined that it was an effective measure for preventing dreadful diseases (Stern and Markel 2005). Late in the eighteenth century Chronicle of Vaccines started. But, in the recent past nineteenth century vaccines were formulated in the research laboratory. Potentially vaccines were developed in the twentieth century based on immune cell markers. However, the advancement of biological engineering in the current century made it possible to develop vaccines that were not possible before (Plotkin 2014).

The term vaccination and immunization are interchangeable, usually comprises a microorganism that is either killed or weakened (Stern and Markel 2005). However, UK Vaccination Acts was passed by the parliament of the UK in different years, but in 1871 another Act was passed particularly when vaccination becomes mandatory in 1852 by appointing a vaccination officer (Allen and Fitzpatrick 2007). In this modernizing era, the anti-vaccination campaign was supported by Alfred Russel Wallace, co-discoverer of evolution (Weber 2010). The foremost human infectious disease smallpox was eradicated after discovering the smallpox vaccine, a milestone achieved in 1979 (Rotz 2009).

Vaccines are made by reducing the virulence of pathogen, but still keeping it viable that induces more effective immunity than killed vaccines. These vaccines are comparatively easier and cheap to create. However, these active vaccines can induce mutations in the attenuated vaccine strain, which reverts to its normal virulent pathogen (Nuttalla and Eley 2011). Alternative ways of making vaccines by using microorganisms are those that were killed and are safe to use. Several vaccines were developed in the late twentieth century based on whole killed organisms which were poorly unsusceptible. In place of introducing a whole-cell vaccine (attenuated or inactivated) into the body, it is better to develop subunit vaccines which were created by using a fragment of the pathogen that elicits a better immune response (Greenwood 2014).

Preventing a disease is far better than treating it. So, developing immunity is the best way of preventing a disease. Immunity is the condition of body’s defence mechanism. Immunity that exists by birth is known as “innate” or “inborn” immunity” which is non-specific. When “foreign invaders” (antigens) invade the human body, the already existed defence system gives rise to proteins (antibodies) to fight against them. Defence system produces specific antibodies to that specific antigen when a child is infected for the first time. This usually takes time as the defence system does not figure out the antigen quickly to fight against getting disease. But for next time when the same antigen enters into the body, immune system recognizes it fast as it already developed a memory cell. So, when the same antigen enters the body after several years, immune system is capable of producing the antibodies fast and keeping it protective from causing disease for the second time which is called as “adaptive” or “acquired” immunity which is antigen specific (Poos et al. 1999).

WHO accomplished the Expanded Programme on Vaccination (EPI) in 1974 in order to intake the vaccines worldwide. This developing world mainly focused in eradicating deadly contagions and dreadful diseases particularly in younger child’s by this vaccination programmes because of high mortality at this age. Progression of vaccines based on their complex structure poses a challenge to the future generation. Vaccines preparation for the diseases like COVID-19 is made easy because of advanced technology (Sabchareon et al. 2012). Demands still exist in development of conventional vaccines. But National Regulatory Authorities (NRAs) are creditworthy to assure the degree, safety and potency of vaccines and other medicinal products. Vaccines undergo different phases of trails before introduction into an immunization programme. After evaluating all the safety assets and efficacy in clinical trials, it undergoes several manufacturing processes where thorough and continuous reviews takes place. NRAs monitor continuously and look into the unpropitious events in vaccination in order to assure that the vaccines are secure to use (Dellepiane and Wood 2015).

### Vaccination—a human responsibility

Vaccination helps in protecting the lives of people when the pandemics like COVID-19 arouses. It is a human responsibility for taking the vaccination in order to protect future generations. There are so many measures to control and respond to pandemics to limit the spread of disease thereby dropping death rate. But having a correspondent vaccine for a particular disease may help in fighting when the pandemics like these arises. However, not all diseases are dreadful, so it is not necessary for having vaccines for all infectious diseases. Following some measures like self-protecting tasks may slowdown the widespread of pandemics, but vaccinums and antiviral narcotics can work effectively in eradicating the transmission rates, thereby treating the sickness. Vaccines cannot be made until or unless the strain causing the disease corresponds to the one that was determined earlier. Vaccines undergo different studies before they enter into the market. Countries will need to have pandemic plans before the pandemic begins. Epidemiological studies help in knowing the transmission modes, transmission rates and the mortality rate in different age groups which helps in designing the vaccines. Currently, vaccines are designed by looking into each age group but mainly to the age that had highest death rates, but this may vary. So, every individual must be vaccinated (Monto 2006). In order to decrease the COVID-19 cases, the preparation of vaccine was also speeded up by the organizations. With the so far available data, the vaccines can be made within a short period of time. When compared to previous year, the data now available are vast and so it is possible for the researchers to prepare various types of vaccines. Different categories of COVID vaccines are in phase III clinical trials. These include protein subunit, whole virus, viral vector, nucleic acid (RNA and DNA). As the COVID-19 cases increase dramatically, several countries also speed up the process of developing vaccines. There were many types of vaccines developed by many countries which were approved by Emergency Use Authorization (EUA). These arrived up or finished phase 3 trails. Many vaccine candidates will be evaluated for safety and efficacy before they got released into the market. Few of them are listed in Tables 2 and 3.

**Table 2:** A list of few pandemics and their epidemiology

Event name	Date	Location	Disease name	Death toll	References
COVID-19 pandemic	2019-present	Worldwide	COVID-19 (SARS-CoV-2 virus)	3,345,085 (as of 14 may 2021)	https://www.who.int/emergencies/diseases/novel-coronavirus-2019/situation-reports
Western African Ebola virus epidemic	2013–2016	Western African Ebola virus epidemic 2013–2016 Worldwide, primarily concentrated in Guinea, Liberia, Sierra Leone	Ebola	11,323+	Gignoux et al. (2015)
2012 Middle East respiratory syndrome coronavirus outbreak	2012-present	Worldwide	Middle East respiratory syndrome / MERS-CoV	935+	http://outbreaknewstoday.com/mers-coronavirus-update-61-cases-reported-in-first-half-of-2020/
2009 Swine flu pandemic	2009–2010	Worldwide	Influenza A virus subtype H1N1	284,000 (possible range 151,700–575,400)	Dawood et al. (2012)
2002–04 SARS outbreak	2002–2004	Worldwide	Severe acute respiratory syndrome (SARS)	774	https://www.who.int/health-topics/severe-acute-respiratory-syndrome
Soviet flu	1977–1978	Worldwide	Influenza A virus subtype H1N1	10,000–30,000	Prieto (2009)
Hong Kong flu	1968–1970	Worldwide	Influenza A virus subtype H3N2	1–4 million	Paul (2003
1957–1958 Influenza pandemic (Asian flu)	1957–1958	Worldwide	Influenza A virus subtype H2N2	1–4 million	Paul (2003)
1918 Influenza pandemic (Spanish flu)	1918–1920	Worldwide	Influenza A virus subtype H1N1	17–100 million	Patterson and Pyle (1991), Spreeuwenberg et al. (2018), Jilani et al. (2020)
1889–1890 flu pandemic	1889–1890	Worldwide	Influenza	1 million	Parsons and Britain (1893)
1520 Mexico Smallpox epidemic	1519–1520	Mexico	Smallpox	5–8 million (40% population)	Acuña-Soto et al. (2002)

**Table 3:** A list of few vaccines that completed clinical trial phase 3

Vaccine name	Research name	Vaccine type	Manufacturer	Country of origin	Side effects	Efficacy	References
Comirnaty	BNT162b2	3 LNP-mRNAs	Pfizer, BioNTech, Fosun Pharma	Multinational	Fatigue, headache, myalgia, chills, arthralgia	95%	Jeff Craven (2021), Kashte et al. (2021)
Moderna COVID-19 vaccine	Mrna-1273	LNP-encapsulated mRNA	Moderna, BARDA, NIAID	US	Tiredness, headache, muscle pain, chills, fever, nausea	94%	Jeff Craven (2021), Kashte et al. (2021)
AstraZeneca also known as Vaxzevria and Covishield	AZD1222	Non-replicating viral vector ChAdOx1-S	BARDA, OWS	UK	Tiredness, headache, muscle pain, chills, fever, nausea	70%	Kashte et al. (2021), Jeff Craven (2021)
Sputnik V		Non-replicating viral vector	Gamaleya research institute, Acellena contract drug research and development	Russia	Breathing difficulties, convulsions, swelling muscle weakness, high BP, headaches, dizziness	92%	Kashte et al. (2021), Jeff Craven (2021)
Janssen	JNJ-78436735; Ad26.COV2.S	Non-replicating viral vector	Janssen vaccines (Johnson & Johnson)	The Netherlands, US	Headache, fever, fatigue, muscle aches, nausea, irritation, redness	68%	Kashte et al. (2021), Jeff Craven (2021)
CoronaVac		Inactivated vaccine (formalin with alum adjuvant)	Sinovac	China	Rash, headache, muscle pain, fever, nausea, vomiting, injection site pain, fatigue	78%	Kashte et al. (2021), Jeff Craven (2021)
Sinopharm	BBIBP-CorV	Inactivated vaccine	Beijing institute of biological products; China national pharmaceutical group (Sinopharm)	China	Lethargy, drowsiness, difficulty sleeping, sneezing, nasopharyngitis, nasal congestion	86%	Kashte et al. (2021), Jeff Craven (2021)
EpiVacCorona		Peptide vaccine	Federal budgetary research institution state research center of virology and biotechnology	Russia	–	–	Jeff Craven (2021)
Convidicea	Ad5-nCoV	Recombinant vaccine (adenovirus type 5 vector)	CanSino Biologics	China	Fever, pain, swelling, sensitivity, fatigue, pain in muscles, joints and heads	66%	Kashte et al. (2021), Jeff Craven (2021)
Covaxin	BBV152	Inactivated vaccine	Bharat Biotech, ICMR; Ocugen	India	Pain, headache, fatigue, fever, body ache, abdominal pain, nausea, tremor, sweating, cold, cough	78%	Kashte et al. (2021), Jeff Craven (2021)
WIBP-CorV		Inactivated vaccine	Wuhan institute of biological products; China national pharmaceutical group (Sinopharm)	China	Rash, headache, muscle pain, fever, nausea, vomiting, injection site pain	72,51%	Jeff Craven (2021)
CoviVac		Inactivated vaccine	Chumakov Federal scientific center for research and development of immune and biological products	Russia	–	–	Jeff Craven (2021)
	ZF2001	Recombinant vaccine	Anhui Zhifei Longcom biopharmaceutical institute of microbiology of the Chinese academy of sciences	China, Uzbekistan	Injection pain, redness, swelling	-	Jeff Craven (2021)
QazVac (QazCovid-in)		Inactivated vaccine	Research institute for biological safety problems	Kazakhstan	–	–	Jeff Craven (2021)
Novavax	NVX-Cov2373	Recombinant nanoparticle	Biofabri in Spain, FUJIFILM Diosynth Biotechnologies (FDB) in both North Carolina and Texas in the US, FDB in the UK, SIIPL in India, SK Bioscience in the Republic of Korea, Takeda Pharmaceutical Company Limited in Japan	UK & South Africa	Rash, headache, muscle pain, fever, nausea, vomiting, injection site pain	89.3%	Novavax (2021)

## Conclusions

We had seen humans have been battling viruses since many years, but we are long way back to fight against them. Many antiviral drugs were used to treat infections years ago. But these advanced technologies made possible to design the vaccines for coronavirus more speedily. Oxford University has already taken the step to discover a safe, effective and accessible vaccine against coronavirus. So, when a pandemic arises, there are difficult decisions ahead for governments. However, we have fundamental knowledge about pandemic risk management, including standard public health measures to protect individuals, and more effective cooperation between medicine and public health. Vaccination is a part public health response to the future emergence of pandemic illness. Though futurity epidemics may not be preventable, we can eradicate them by enforcing public prevention measures, slowing pandemic viral spread or vaccine manufacture and seasonal decreases in transmission. We have developed vaccines that can prevent pandemics and our current discoveries will save millions of lives in future. The pandemic is still continuing, and information furnished is to an extent known till date; worldwide countries are trying to develop novel therapeutics and vaccines to mitigate the virus and its rigorous forms caused by mutations. Globally, research findings emerge with new findings to curb the current death troll and prevent upcoming waves to bring back the world to a better place post-pandemic.

## Data Availability

Not Applicable.

## Abbreviations

SARS-CoV-2: Severe acute respiratory syndrome coronavirus 2
COVID-19: Coronavirus disease 2019
WHO: World Health Organisation
MERS: Middle East respiratory syndrome
SARS: Severe acute respiratory syndrome
ICTV: International Committee on Taxonomy of Viruses
ACE2: Angiotensin-converting enzyme receptor 2
TMPRSS2: Transmembrane serine protease 2
ERGIC: Endoplasmic reticulum-Golgi intermediate compartment
EVD: Ebola viral disease
NRA: National Regulatory Authorities (NRAs)

## Declarations

**Ethical approval and consent to participate.**

Not applicable.

## References

[CR1] Acuña-Soto R, Stahle DW, Cleaveland MK, Therrell MD (2002). Megadrought and megadeath in 16th century Mexico. Emerg Infect Dis.

[CR500] Allen A, Fitzpatrick M (2007) Vaccine: the controversial story of medicine's greatest lifesaver. J Royal Soc Med 100(5):241–241

[CR2] Annavajhala MK, Mohri H, Zucker JE (2021) A Novel SARS-CoV-2 Variant of Concern, B.1.526, Identified in New York. MedRxiv

[CR3] Artika IM, Dewantari AK, Wiyatno A (2020) Molecular biology of coronaviruses: current knowledge. Heliyon, p e04743 10.1016/j.heliyon.2020.e04743 PMC7430346 32835122

[CR4] Bar-On YM, Flamholz A, Phillips R, Milo R (2020). Science Forum: SARS-CoV-2 (COVID-19) by the numbers. Elife.

[CR5] Belouzard S, Millet JK, Licitra BN, Whittaker GR (2012). Mechanisms of coronavirus cell entry mediated by the viral spike protein. Viruses.

[CR6] Bosch BJ, Van der Zee R, De Haan CA, Rottier PJ (2003). The coronavirus spike protein is a class I virus fusion protein: structural and functional characterization of the fusion core complex. J Virol.

[CR7] Bosch BJ, De Haan CA, Smits SL, Rottier PJ (2005). Spike protein assembly into the coronavirion: exploring the limits of its sequence requirements. Virology.

[CR8] Cherian S, Potdar V, Jadhav S, Yadav P, Gupta N, Das M, Das S, Agarwal A, Singh S, Abraham P, Panda S (2021) Convergent evolution of SARS-CoV-2 spike mutations, L452R, E484Q and P681R, in the second wave of COVID-19 in Maharashtra, India. bioRxiv 10.3390/microorganisms9071542 PMC8307577 34361977

[CR9] Dandagi GL, Byahatti SM (2011). An insight into the swine-influenza A (H1N1) virus infection in humans. Lung India.

[CR10] Dawood FS, Iuliano AD, Reed C, Meltzer MI, Shay DK, Cheng PY, Bandaranayake D, Breiman RF, Brooks WA, Buchy P, Feikin DR (2012). Estimated global mortality associated with the first 12 months of 2009 pandemic influenza A H1N1 virus circulation: a modelling study. Lancet Infect Dis.

[CR11] De Haan CA, Rottier PJ (2005). Molecular interactions in the assembly of coronaviruses. Adv Virus Res.

[CR13] De Haan CA, Vennema H, Rottier PJ (2000). Assembly of the coronavirus envelope: homotypic interactions between the M proteins. J Virol.

[CR12] De Haan CA, Smeets M, Vernooij F, Vennema H, Rottier PJ (2020). Mapping of the coronavirus membrane protein domains involved in interaction with the spike protein. J Virol.

[CR14] Dellepiane N, Wood D (2015). Twenty-five years of the WHO vaccines prequalification programme (1987–2012): lessons learned and future perspectives. Vaccine.

[CR15] Elbe S, Buckland-Merrett G (2017). Data, disease and diplomacy: GISAID's innovative contribution to global health. Global Chall.

[CR16] Gaurav A, Al-Nema M (2019) Polymerases of coronaviruses: structure, function, and inhibitors. In: Viral polymerases. Academic Press, New York, pp 271–300

[CR17] Gibbs AJ, Armstrong JS, Downie JC (2009). From where did the 2009 swine origin influenza A virus (H1N1) emerge?. Virol J.

[CR18] Gignoux E, Idowu R, Bawo L, Hurum L, Sprecher A, Bastard M, Porten K (2015). Use of capture–recapture to estimate underreporting of Ebola virus disease, Montserrado County. Liberia Emerg Infect Dis.

[CR19] Greenwood B (2014). The contribution of vaccination to global health: past, present and future. Philos Trans R Soc B Biol Sci.

[CR20] Harapan H, Itoh N, Yufika A, Winardi W, Keam S, Te H, Megawati D, Hayati Z, Wagner AL, Mudatsir M (2020). Coronavirus disease 2019 (COVID-19): a literature review. J Infect Public Health 13(5):667–673 10.1016/j.jiph.2020.03.019 PMC7142680 32340833

[CR21] Hoffmann M, Kleine-Weber H, Schroeder S, Krüger N, Herrler T, Erichsen S, Schiergens TS, Herrler G, Wu NH, Nitsche A, Müller MA (2020). SARS-CoV-2 cell entry depends on ACE2 and TMPRSS2 and is blocked by a clinically proven protease inhibitor. Cell.

[CR22] Hogue BG, Machamer CE (2007). Coronavirus structural proteins and virus assembly. Nidoviruses.

[CR23] http://outbreaknewstoday.com/mers-coronavirus-update-61-cases-reported-in-first-half-of-2020/

[CR24] https://www.who.int/emergencies/diseases/novel-coronavirus-2019/situation-reports

[CR25] https://www.who.int/health-topics/severe-acute-respiratory-syndrome

[CR26] Hui DS, Azhar EI, Madani TA, Ntoumi F, Kock R, Dar O, Ippolito G, Mchugh TD, Memish ZA, Drosten C, Zuml A (2020). The continuing 2019-nCoV epidemic threat of novel coronaviruses to global health—The latest 2019 novel coronavirus outbreak in Wuhan, China. Int J Infect Dis.

[CR27] Jeff Craven (2021) COVID-19 vaccine tracker. https://www.raps.org/news-and-articles/news-articles/2020/3/covid-19-vaccine-tracker

[CR28] Jilani TN, Jamil RT, Siddiqui AH (2020) H1N1 influenza (swine flu). StatPearls [Internet].

[CR29] Jones KE, Patel NG, Levy MA, Storeygard A, Balk D, Gittleman JL, Daszak P (2008). Global trends in emerging infectious diseases. Nature.

[CR30] Jordan WS (1961). The mechanism of spread of Asian influenza. Am Rev Respir Dis.

[CR31] Kashte S, Gulbake A, El-Amin SF, Gupta A (2021). COVID-19 vaccines: rapid development, implications, challenges and future prospects. Hum Cell.

[CR32] Kourtis AP, Appelgren K, Chevalier MS, McElroy A (2015). Ebola virus disease: focus on children. Pediatr Infect Dis.

[CR33] Lan J, Ge J, Yu J, Shan S, Zhou H, Fan S, Zhang Q, Shi X, Wang Q, Zhang L, Wang X (2020). Structure of the SARS-CoV-2 spike receptor-binding domain bound to the ACE2 receptor. Nature.

[CR34] Li Q, Med M, Xuhua G, Peng W, Xiaoye W (2020). Early transmission dynamics in Wuhan, China, of novel coronavirus-Infected Pneumonia. N Engl J Med.

[CR35] Li X, Geng M, Peng Y, Meng L, Lu S (2020). Molecular immune pathogenesis and diagnosis of COVID-19. J Pharm Anal.

[CR36] Li LH (2004). Epidemiologic clues to SARS origin in China. Emerg Infect Dis.

[CR37] Liu P, Jiang JZ, Wan XF, Hua Y, Li L, Zhou J, Wang X, Hou F, Chen J, Zou J, Chen J (2020). Are pangolins the intermediate host of the 2019 novel coronavirus (SARS-CoV-2). PLoS Pathog.

[CR38] Lotfi M, Hamblin MR, Rezaei N (2020). COVID-19: Transmission, prevention, and potential therapeutic opportunities. Clin Chim Acta.

[CR39] Martini M, Gazzaniga V, Bragazzi NL, Barberis I (2019). The Spanish Influenza Pandemic: a lesson from history 100 years after 1918. J Prev Med Hyg.

[CR40] Masters PS (2006). The molecular biology of coronaviruses. Adv Virus Res.

[CR41] McBride R, Van Zyl M, Fielding BC (2014). The coronavirus nucleocapsid is a multifunctional protein. Viruses.

[CR42] Millet JK, Whittaker GR (2015). Host cell proteases: critical determinants of coronavirus tropism and pathogenesis. Virus Res.

[CR43] Monto AS (2006). Vaccines and antiviral drugs in pandemic preparedness. Emerg Infect Dis.

[CR44] Morse SS (2001). Factors in the emergence of infectious diseases. Emerg Infect Dis.

[CR45] Murphy F (1998). Emerging Zoonoses. Emerg Infect Dis.

[CR46] Narayanan K, Chen CJ, Maeda J, Makino S (2003). Nucleocapsid-independent specific viral RNA packaging via viral envelope protein and viral RNA signal. J Virol.

[CR47] Neuman BW, Joseph JS, Saikatendu KS, Serrano P, Chatterjee A, Johnson MA, Liao L, Klaus JP, Yates JR, Wüthrich K, Stevens RC (2008). Proteomics analysis unravels the functional repertoire of coronavirus nonstructural protein 3. J Virol.

[CR48] Neuman BW, Kiss G, Kunding AH, Bhella D, Baksh MF, Connelly S, Droese B, Klaus JP, Makino S, Sawicki SG, Siddell SG (2011). A structural analysis of M protein in coronavirus assembly and morphology. J Struct Biol.

[CR49] Novavax (2021). Press release. https://ir.novavax.com/news-releases/news-release-details/novavax-covid-19-vaccine-demonstrates-893-efficacy-uk-phase-3

[CR50] Nuttala JJ, Eley BS (2011) BCG vaccination in HIV-infected children. Tuberculosis Res Treat 2011:712736. 10.1155/2011/712736 10.1155/2011/712736 PMC3335547 22567268

[CR51] Ortega JT, Serrano ML, Pujol FH, Rangel HR (2020). Role of changes in SARS-CoV-2 spike protein in the interaction with the human ACE2 receptor: an in silico analysis. EXCLI J.

[CR52] Parsons HF, Britain G (1893). Further report and papers on epidemic influenza, 1889–92: with an introduction by the Medical Officer of the Local Government Board. HM Stationery Office

[CR53] Patterson KD, Pyle GF (1991). The geography and mortality of the 1918 influenza pandemic. Bull His Med.

[CR54] Paul WE (2003). Fundamental immunology.

[CR55] Perlman S, Netland J (2009). Coronaviruses post-SARS: update on replication and pathogenesis. Nat Rev Microbiol.

[CR56] Plotkin S (2014). History of vaccination. Proc Natl Acad Sci.

[CR57] Poos MI, Costello R, Carlson-Newberry SJ (1999) Institute of Medicine (US) Committee on Military Nutrition Research. Committee on Military Nutrition Research: Activity Report: December 1, 1994 through May 31, 1999. National Academies Press (US), Washington (DC), Military Strategies for Sustainment of Nutrition and Immune Function in the Field 25077229

[CR58] Porta M (ed) (2014) A dictionary of epidemiology. Oxford university press. ISBN: 978-0-19-997673-7

[CR59] Prieto JR (2009). Las pandemias de la gripe. Rev Esp Quimioter.

[CR60] Ramadan N, Shaib H (2019). Middle East respiratory syndrome coronavirus (MERS-CoV): review. Germs.

[CR61] Raoult D, Zumla A, Locatelli F, Ippolito G, Kroemer G (2020). Coronavirus infections: epidemiological, clinical and immunological features and hypotheses. Cell Stress.

[CR62] Rota PA, Oberste MS, Monroe SS, Nix WA, Campagnoli R, Icenogle JP, Penaranda S, Bankamp B, Maher K, Chen MH, Tong S (2003). Characterization of a novel coronavirus associated with severe acute respiratory syndrome. Science.

[CR63] Rotz L (2009). Smallpox—the death of a disease: The inside story of eradicating a worldwide killer. J Clin Investig.

[CR64] Sabchareon A, Wallace D, Sirivichayakul C, Limkittikul K, Chanthavanich P, Suvannadabba S, Jiwariyavej V, Dulyachai W, Pengsaa K, Wartel TA, Moureau A (2012). Protective efficacy of the recombinant, live-attenuated, CYD tetravalent dengue vaccine in Thai schoolchildren: a randomised, controlled phase 2b trial. Lancet.

[CR65] Shang J, Ye G, Shi K (2020). Structural basis of receptor recognition by SARS-CoV-2. Nature.

[CR66] Silverman RH (2020). COVID-19: Coronavirus replication, pathogenesis, and therapeutic strategies. Clevel Clin J Med.

[CR67] Siu YL, Teoh KT, Lo J, Chan CM, Kien F, Escriou N, Tsao SW, Nicholls JM, Altmeyer R, Peiris JS, Bruzzone R (2008). The M, E, and N structural proteins of the severe acute respiratory syndrome coronavirus are required for efficient assembly, trafficking, and release of virus-like particles. J Virol.

[CR68] Spreeuwenberg P, Kroneman M, Paget J (2018). Reassessing the global mortality burden of the 1918 influenza pandemic. Am J Epidemiol.

[CR69] Stern AM, Markel H (2005). The history of vaccines and immunization: familiar patterns, new challenges. Health Aff.

[CR70] Surjit M, Lal SK (2008). The SARS-CoV nucleocapsid protein: a protein with multifarious activities. Infect Genet Evol.

[CR71] Tang X, Wu C, Li X, Song Y, Yao X (2020). On the origin and continuing evolution of SARS-CoV-2. Natl Sci Rev.

[CR72] Tiwari R, Dhama K, Sharun K, Iqbal Yatoo M, Malik YS, Singh R, Michalak I, Sah R, Bonilla-Aldana DK, Rodriguez-Morales AJ (2020). COVID-19: animals, veterinary and zoonotic links. Vet Q.

[CR73] Venkatagopalan P, Daskalova SM, Lopez LA, Dolezal KA, Hogue BG (2015). Coronavirus envelope (E) protein remains at the site of assembly. Virology.

[CR74] Wang P, Nair MS, Liu L, Iketani, Luo Y, Guo Y, Wang M, Yu J, Zhang B, Kwong PD, Graham BS (2021) Antibody resistance of SARS-CoV-2 variants B. 1.351 and B. 1.1. 7. Nature 1–6 10.1038/s41586-021-03398-2 33684923

[CR75] Weber TP (2010). Alfred Russel Wallace and the antivaccination movement in Victorian England. Emerg Infect Dis.

[CR76] Wilson L, Mckinlay C, Gage P, Ewart G (2004). SARS coronavirus E protein forms cation-selective ion channels. Virology.

[CR77] Woolhouse ME, Gowtage-Sequeria S (2005). Host range and emerging and reemerging pathogens. Emerg Infect Dis.

[CR78] Wu K, Werner AP, Moliva JI, Koch M, Choi A, Stewart-Jones GB, Bennett H, Boyoglu-Barnum S, Shi W, Graham BS, Carfi A (2021) mRNA-1273 vaccine induces neutralizing antibodies against spike mutants from global SARS-CoV-2 variants. BioRxiv

[CR79] Xu RH, He JF, Evans MR, Peng GW, Field HE, Yu DW, Lee CK, Luo HM, Lin WS, Lin P, Li LH, Liang WJ, Lin JY, Schnur A (2004). Epidemiologic Clues to SARS Origin in China. Emerg Infect Dis.

[CR501] Yadav P, Sapkal GN, Abraham P, Ella R, Deshpande G, Patil DY, Nyayanit D, Gupta N, Sahay RR, Shete AM, Panda S, Bhargava B, Krishna Mohan V (2021) Neutralization of variant under investigation B. 1.617 with sera of BBV152 vaccinees. Clin Infect Dis. 10.1093/cid/ciab411 10.1093/cid/ciab411 33961693

[CR80] Yuki K, Fujiogi M, Koutsogiannaki S (2000) COVID-19 pathophysiology: a review. Clin Immunol 108427 10.1016/j.clim.2020.108427 PMC7169933 32325252

[CR81] Zhou B, Thao TTN, Hoffmann D, Taddeo A, Ebert N, Labroussaa F, Pohlmann A, King J, Steiner S, Kelly JN, Portmann J (2021). SARS-CoV-2 spike D614G change enhances replication and transmission. Nature.

[CR82] Zhou Y, Hou Y, Shen J, Huang Y, Martin W, Cheng F (2020). Network-based drug repurposing for novel coronavirus 2019-nCoV/SARS-CoV-2. Cell Discov.

